# Supplementary material for: Characterization and Expression of Holothurian Wnt Signaling Genes during Adult Intestinal Organogenesis
Source: Genes (Basel). 2023 Jan 25;14(2):309. doi: 10.3390/genes14020309 (PMC9957329; doi:10.3390/genes14020309)
Supplement: Supplementary file 1 [file genes-14-00309-s001.zip › genes-2133551-supplementary.pdf]

Table S1. Wnt Genes Characterization through NCBI Basic Local Alignment.

Wnt	Match Species	Accesion	% Identity	E-Value
Wnt1	*L. variegatus*	MK029663.1	68.19	4e-67
Wnt2	*E. fraudatrix*	MK318552.1	74.92	3e-177
Wnt3	*E. fraudatrix*	MK318553.1	71.60	2e-135
Wnt4a	*E. fraudatrix*	KU061282.2	67.83	2e-83
Wnt4b	*E. fraudatrix*	KU061282.2	72.17	8e-146
Wnt5	*H. leucospilota*	AB969706.1	96.63	0.0
Wnt6	*A. japonicus*	JQ753331.1	76.18	0.0
Wnt7	*E. fraudatrix*	MK318555.1	73.12	1e-155
Wnt9	*E. fraudatrix*	MK318557.1	66.49	2e-59
Wnt10	*A. planci*	XM_022242684.1	75.16	2e-15
Wnt16	*E. fraudatrix*	KT362220.1	70.00	5e-10
WntA	*A. japonicus*	KU888892.1	77.46	0.0

Table S2. NCBI Accession of Wnt Sequences of Distinct Echinoderms.

Species	Wnt	NCBIAccession	Comments
	Wnt1	XP_041475650.1	
	Wnt3	XP_041478374.1	
	Wnt4	XP_041479642.1	
	Wnt5b	XP_041460654.1	
	Wnt6	XP_041475661.1	
L. variegatus	Wnt7b	XP_041459365.1	
	Wnt8a	XP_041485541.1	
	Wnt9a	XP_041475638.1	
	Wnt10b	XP_041477044.1	
	Wnt16	XP_041457833.1	
	WntA	XP_041464773.1	Appears as Wnt1-like
	Wnt1	XP_038047182.1	
	Wnt2	XP_038054103.1	
	Wnt3	XP_038073218.1	
	Wnt4	XP_038061751.1	
	Wnt5b	XP_038054542.1	
	Wnt6	XP_038047180.1	
P. miniata	Wnt7b	XP_038069369.1	Appears as an isoform of Wnt7
	Wnt8	XP_038059603.1	
	Wnt9	XP_038047188.1	
	Wnt10b	XP_038047890.1	
	Wnt11	XP_038051134.1	
	Wnt16	XP_038054431.1	
	WntA	XP_038076029.1	
	Wnt1	NP_001116972.1	
	Wnt3	XP_030830597.1	
	Wnt4	XP_030842454.1	
	Wnt5b	XP_011670176.1	
	Wnt6	XP_790077.1	
S. purpuratus	Wnt7b	XP_787051.3	
	Wnt8	NP_999832.1	
	Wnt9	XP_030830874.1	
	Wnt10	XP_011664244.1	
	Wnt16	XP_796616.2	
	WntA	XP_030832838.1	Appears as Wnt7a
	Wnt2	PIK43830.1	
	Wnt3	PIK62708.1	
	Wnt4	PIK52961.1	
	Wnt5	PIK40288.1	
	Wnt6	AGA62464.1	
A. japonicus	Wnt7A	PIK56278.1	
	Wnt7B	PIK48022.1	
	Wnt8A	PIK51024.1	
	Wnt8B	PIK51023.1	
	Wnt9	PIK51469.1	
	WntA	PIK57158.1	
	Wnt2	QEF51147.1	
	Wnt3	QEF51148.1	
	Wnt5	QEF51149.1	
	Wnt7	QEF51150.1	
E. fraudatrix	Wnt8	QEF51151.1	
	Wnt9	QEF51152.1	
	Wnt10	QDW65356.1	
	Wnt16	ALT56982.1	
	WntA	QDW65349.1	

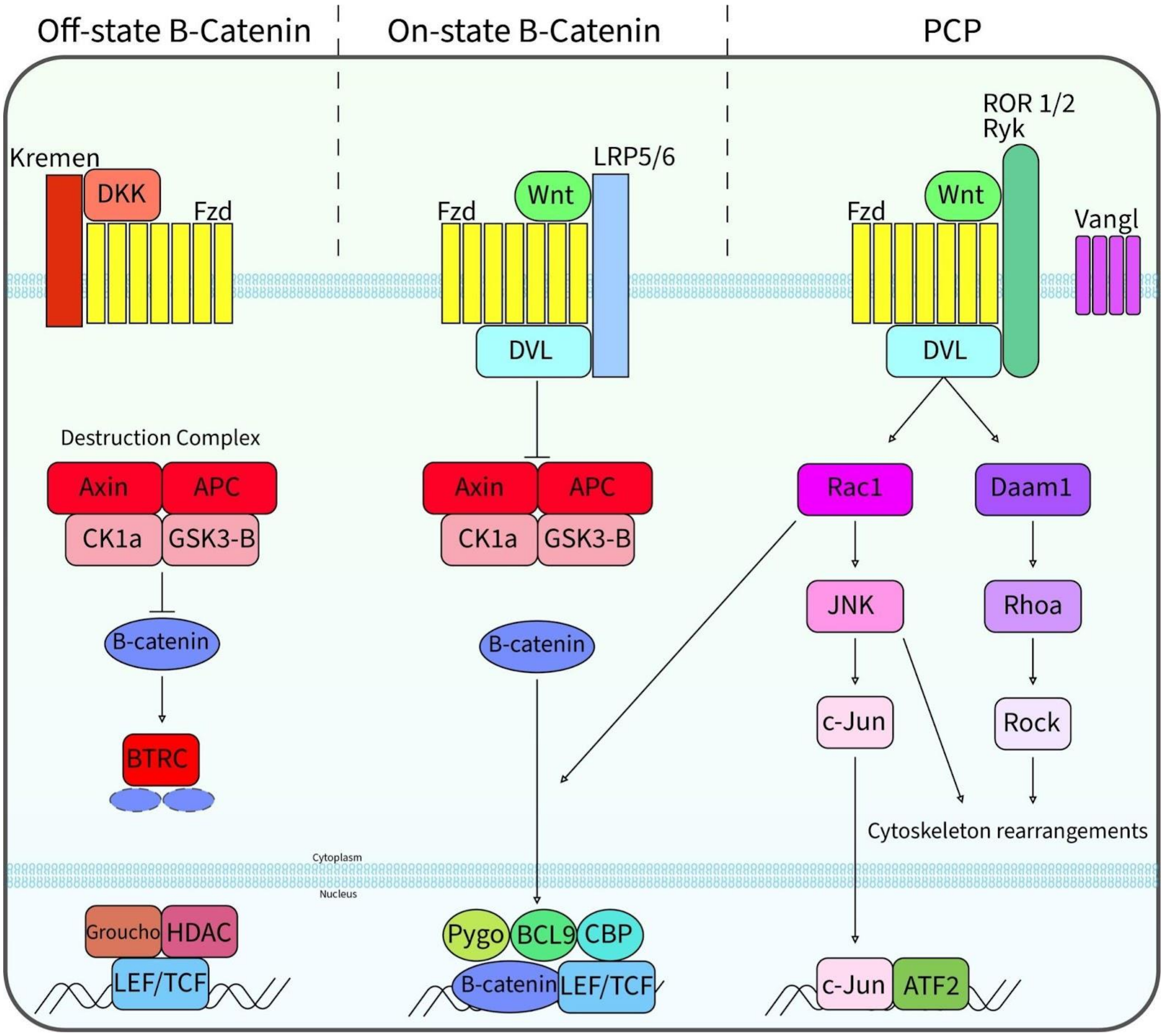

Figure S1. Brief overview of the signaling genes in the Wnt/β-catenin pathway and the Wnt/PCP pathway. Wnt/β-catenin has an Off- and On-state. When Wnt/β-catenin is in the Off-state, Kremen and DKK prevent Wnt from binding Fzd while β-catenin is labeled for proteasomal degradation by the destruction complex and in the nucleus the LEF/TCF transcription factor is inhibited by the corepressors Groucho and HDAC. In the On-state, Wnt binds Fzd and recruits the co-receptor LRP5/6, then DVl attaches and inhibits the destruction complex, allowing β-catenin to translocate in the nucleus where it binds to LEC/TCF and to other co-activators. One of the downstream effects of the Wnt/PCP is the recruitment of Vangl to the cell-membrane which can alter cell polarity. Although these pathways are distinct, there is crosstalk between them. For example, RAC1 has been shown to help translocate β-catenin into the nucleus, although being a part of Wnt/PCP pathway [7]. Additionally, Vangl can inhibit canonical Wnt signaling [11].

```
Score             Expect   Identities        Gaps           Strand
1256 bits(680)    0.0      920/1039(89%)     3/1039(0%)     Plus/Plus

Query  1     ATGCGGGTGGCAACGTGTATACATCTTGTTTTAACGATTGTATTTATCATTACATTGAAC  60
Sbjct  1     ...T...C.A....C....C...............A...C.T..C..........C.T..  60

Query  61    TGTGTTCAGGTCAACTATGCGTGGTTGTCGATGTTCAACATTGAAGCTATAGGAATAAAC  120
Sbjct  61    .......GA......C....T.....................C.................  120

Query  121   TCTATTGAAAACAATGAAACTTGCGAAATTATTCCAGGACTCGTAAACAGACAGGTTGTG  180
Sbjct  121   .............CA.............................G.....G..A..G.C.  180

Query  181   ATTTGTAAGAGAAACTTGGAGGTGATGGACAGTGTAAGCAACGGGGCATCCATTGCCATT  240
Sbjct  181   .......................A...........T.....A............T.....  240

Query  241   CTCGAGTGTCAGGCACAGTTCCAGTACAGGAGGTGGAATTGTTCAATAGTTGATCCGTAT  300
Sbjct  241   ...........AAA.........CA....A................C.............  300

Query  301   ACCGTCTTCGGACCGGTCCTTGACAGTGGAACCAGAGAGGCAGCGTTTGTGAGTTCTATT  360
Sbjct  301   ....................C..T.AC........G.....................G..  360

Query  361   ACCGCGGCTGGAGTGGTGCACGCCGTGACGCGTTCCTGCAGCCTTGGCGAACTTTCGAAG  420
Sbjct  361   ................C............A.................G..G....T....  420

Query  421   TGCGGTTGTGATCGAACGTTATCCGGTATTAGTCCAGACGGTTTTATGTGGTCGGGGTGT  480
Sbjct  421   ..T...............G.........C.........A.......C.............  480

Query  481   TCCGATGATGTGGCCTATGGTATCAGGTTTTCAAGAAAGTTTGTCGACGCTAGTGAATCG  540
Sbjct  481   ......A.................CA........A..T...C...........A.....A  540

Query  541   ACATATAGGGTCTCTATCGCCAGGCGACTAATGAATTTACACAACAACAGAGCTGGGAGA  600
Sbjct  541   .A..CC.............A...A....................T...GA..........  600

Query  601   AGGGTCATCAAAGATCACATGAAGCTTGGATGCAAATGCCATGGCATTTCTGGTTCGTGT  660
Sbjct  601   ....C...TG.....A.......AAC..A............C...G..............  660

Query  661   GAAGTTCGATCCTGTTGGAGGTCATTGCCTTCCTTCAAGAGGGTTGGTAGTGTACTCAAA  720
Sbjct  661   ......AA..T.............A............G...........C..........  720

Query  721   ACGAAATTTGACGACGCAACAAAAGTAGCCCGACAGAACATCAGCTCTCGTCAACAACTC  780
Sbjct  721   GA...........G.......G.......T.A...A.GA.............G.......  780

Query  781   GTGCCAGTCAATCCCCACTTTGGACCCCATACGAATTCTGATTTGGTATACTTGAAGAAT  840
Sbjct  781   ..............G..T.A.AA.........A.....................C.....  840

Query  841   TCTCCAAATTTTTGCGAGAGAAATCTTTCGATCGGGTCTCTTGGAACGGAAAATAGGACA  900
Sbjct  841   ......G....................G....T...............C....C......  900

Query  901   TGCAACAAAGATTCTAAAGCCATCGACAGCTGCGAGCTTCTCTGCTGTGGACGTGGCTAC  960
Sbjct  901   ....................T......G................................  960

Query  961   CATACAAAAAAACCAAACGATCAGCGAACAATGCATGTGCAAATTTTATTGGTGCT--TTA  1018
Sbjct  961   A.........T.G....CG.........G................C.........GCG.T  1020

Query  1019  GG-AAATGCAAAATATGTA  1036
Sbjct  1021  .TC................  1039
```

Supplemental Figure S2. NCBI Blast Alignment of Wnt4b against Wnt4a. Both genes were found in the transcriptome. To ensure that the transcripts were distinct and not a sequencing error, a sequence alignment was made. The query is Wnt4b and the subject is Wnt4a. Both genes were of similar length and displayed an 89% identity with only 3 gaps in the sequence alignment.

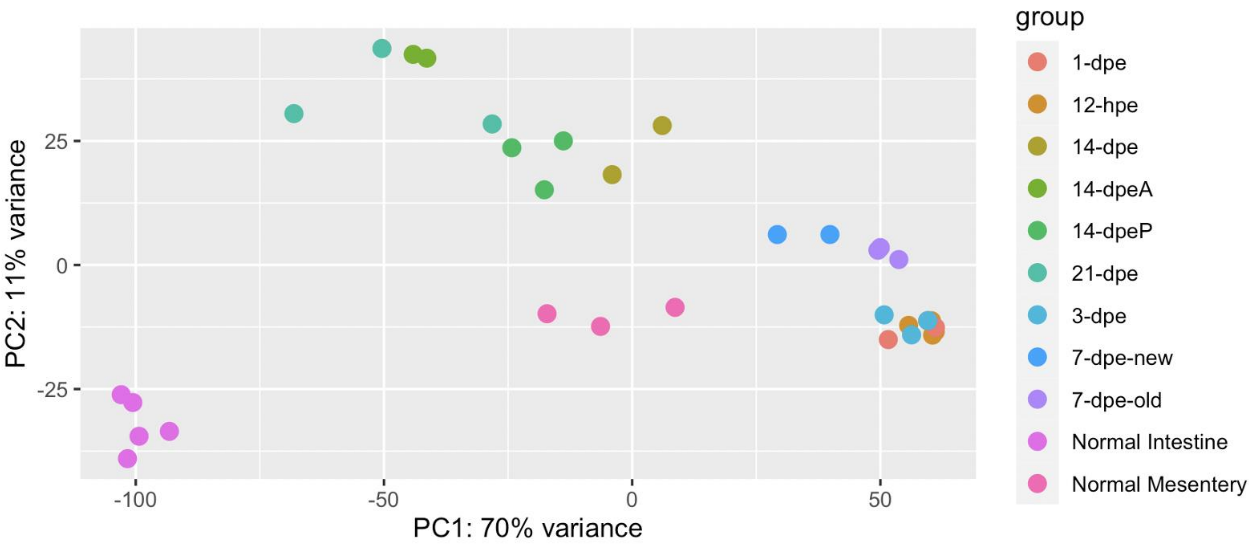

Figure S3. PCA plot of sequencing files. All timepoints that have accumulated in the *H. glaberrima* intestinal transcriptomic database. Each dot in a group on the plot represents a sample that was pooled, containing the intestine or mesentery of at least two different sea cucumbers.
